# A Timeframe for SARS-CoV-2 Genomes: A Proof of Concept for Postmortem Interval Estimations

**DOI:** 10.3390/ijms232112899

**Published:** 2022-10-25

**Authors:** Jacobo Pardo-Seco, Xabier Bello, Alberto Gómez-Carballa, Federico Martinón-Torres, José Ignacio Muñoz-Barús, Antonio Salas

**Affiliations:** 1 Grupo de Investigacion en Genética, Vacunas, Infecciones y Pediatría (GENVIP), Hospital Clínico Universitario, Universidade de Santiago de Compostela, 15706 Santiago de Compostela, Galicia, Spain; 2 GenPoB Research Group, Instituto de Investigación Sanitaria (IDIS), Hospital Clínico Universitario de Santiago (SERGAS), 15706 Santiago de Compostela, Galicia, Spain; 3 Unidade de Xenética, Instituto de Ciencias Forenses (INCIFOR), Facultade de Medicina, Universidade de Santiago de Compostela, 15705 Santiago de Compostela, Galicia, Spain; 4 Centro de Investigación Biomédica en Red de Enfermedades Respiratorias, Instituto de Salud Carlos III, 28029 Madrid, Comunidad de Madrid, Spain; 5 Translational Pediatrics and Infectious Diseases, Department of Pediatrics, Hospital Clínico Universitario de Santiago de Compostela, 15706 Santiago de Compostela, Galicia, Spain; 6 Department of Forensic Sciences, Pathology, Gynaecology and Obstetrics and Paediatrics, Universidade de Santiago de Compostela, 15705 Santiago de Compostela, Galicia, Spain; 7 Institute of Forensic Sciences (INCIFOR), Universidade de Santiago de Compostela, 15706 Santiago de Compostela, Galicia, Spain

**Keywords:** SARS-CoV-2, phylogeny, legal medicine, molecular clock, postmortem interval, forensic genetics

## Abstract

Establishing the timeframe when a particular virus was circulating in a population could be useful in several areas of biomedical research, including microbiology and legal medicine. Using simulations, we demonstrate that the circulation timeframe of an unknown SARS-CoV-2 genome in a population (hereafter, estimated time of a queried genome [QG]; *t*_E-QG_) can be easily predicted using a phylogenetic model based on a robust reference genome database of the virus, and information on their sampling dates. We evaluate several phylogeny-based approaches, including modeling evolutionary (substitution) rates of the SARS-CoV-2 genome (~10^−3^ substitutions/nucleotide/year) and the mutational (substitutions) differences separating the QGs from the reference genomes (RGs) in the database. Owing to the mutational characteristics of the virus, the present Viral Molecular Clock Dating (VMCD) method covers timeframes going backwards from about a month in the past. The method has very low errors associated to the *t*_E-QG_ estimates and narrow intervals of *t*_E-QG_, both ranging from a few days to a few weeks regardless of the mathematical model used. The SARS-CoV-2 model represents a proof of concept that can be extrapolated to any other microorganism, provided that a robust genome sequence database is available. Besides obvious applications in epidemiology and microbiology investigations, there are several contexts in forensic casework where estimating *t*_E-QG_ could be useful, including estimation of the postmortem intervals (PMI) and the dating of samples stored in hospital settings.

## 1. Introduction

The Postmortem Interval (PMI) is defined as the time elapsed after death. Estimating the PMI is one of the most challenging issues in forensic sciences and has a profound impact in legal medicine. Among the traditional methods used to determine the PMI are those based on the examination of the body (post-mortem changes, e.g., cooling after death, rigor mortis, decomposition changes occurring after death), analytical techniques (e.g., vitreous humor changes), or forensic entomology (e.g., analyzing the life cycle of insects colonizing the body). New molecular biology procedures based on the time-dependent degradation of biological markers have been developed in the last few years, with a special focus on the analysis of RNA [1,2]. In the last decade, dozens of contributions have appeared on the analysis of endogenous reference genes in different biological samples, aimed at analyzing degradation of post-mortem RNA and considering external parameters such as time, cause of death, environmental conditions, etc. (reviewed in [2]). In this line, hope for better procedures has risen from the analysis of microRNAs as housekeeping genes, due to their postmortem stability and resistance to degradation [3]. Other recent developments in genomic sciences have opened new avenues for the determination of the PMI, including exploring the microbiome of dead bodies [4,5]. Also, the analysis of the thanatomicrobiome (i.e., the microbial community associated with the host after death) has emerged as a new tool for the estimation of the PMI, by exploring the microbial succession associated with skin, bone, and soil, taking place in and around corpses during decomposition [6]. All these methods are generally used to estimate PMI in early stages of decomposition and are very sensitive to environmental conditions at the death scene, and therefore undesirably inaccurate. Qualitative anthropological methods are the only solution for PMI periods longer than a month since death, but there are no approved standards. Most studies of longer PMIs are based on the observation of the sequence of stages of decay and are strongly dependent on environmental conditions [7].

Here, we propose that dating the genome of a microorganism obtained from a corpse or a body tissue stored in a hospital may serve the purpose of pointing to a moment in the past (ranging from about a month in the past to years before death, or when a biological sample of interest was stored in, e.g., a hospital) with narrow error estimates (of only a few days/weeks). This approach might open new perspectives in the field of legal medicine, assisting in the resolution of questions related to PMI or time of sample collection from a tissue infected by a pathogen, and covering a period of the PMI for which no accepted quantitative methods are available. The method developed in the present study is based on phylogenetics and takes advantage of the molecular clock represented by the viral RNA. There are multiple methods to date a node in a phylogenetic tree, but usually these methods are computationally very demanding (especially when considering large sequence datasets) and time-consuming. For example, the popular tool BEAST requires days or weeks to run datasets of only a few hundreds of sequences [8]. In addition, these methods require highly specialized personnel to manage multiple parameters related to complex evolutionary theory. Phylogenetic methods for dating are ultimately based on the molecular clock concept. This term refers to the use of the almost constant mutation rate of biomolecules (preferably DNA) to infer the time in the past related to an organism [9]; see examples in humans [10,11,12,13]. When applied to microorganisms, it is possible to estimate e.g., the Time of the Most Recent Common Ancestor (TMRCA) for a given genome, the time of divergence, and the time of a viral outbreak, as recently carried out with the human severe acute respiratory syndrome coronavirus 2 (SARS-CoV-2) [14,15,16], which ultimately led to the coronavirus disease or COVID-19 and was responsible for the pandemic initiated at the end of 2019 [17]. Given the high evolutionary rate of a RNA virus (in the order of 10^−3^ substitutions/nucleotide/year) and its fast replication cycles of a few hours [18], a given viral genome circulating in a population can only last a short time period of a few weeks. After this time, it is expected that this genome will have evolved into new derivative sequences by accumulating mutational changes at a somehow predictable rate (according to its molecular clock). Therefore, taking advantage of this evolutionary molecular clock, it is possible to estimate the time range when a particular viral genome was circulating in a population.

Here, we explore a new rapid and simple approach to date the genome of a microorganism. The present Viral Molecular Clock Dating (VMCD) method is computationally fast and can be implemented without specialized knowledge of evolutionary theory despite being based in phylogenetics. The COVID-19 pandemic has set the ground to explore new phylogenetic techniques given the availability of several million genome sequences produced by public health agencies and governments worldwide to keep track of circulating SARS-CoV-2 variants and understand infection patterns [19,20,21,22].

Thus, we can theorize about a scenario where the RNA material of a coronavirus can be retrieved from, e.g., corpses or human remains, or biopsy samples stored in hospitals, and fully genome sequenced, and from which the estimation of the date this specific viral genome was circulating in the general population could be of interest in a legal medicine context. Given the short life span of a viral genome in a population (marked by the unavoidable course of its RNA molecular evolution), its dating could be used as a timescale proxy for, e.g., a narrow PMI (of only a few days or weeks) of both recent and very old samples, significantly improving traditional approaches.

The present study aims at developing a method to estimate the timeframe of circulation of a SARS-CoV-2 genome in a population using a large genome database as reference, and taking advantage of the metadata available for these reference genomes, including date of collection of the viral samples. The potential interest of this technique is then discussed with a special focus on PMI estimations.

## 2. Results

### 2.1. Notes on the Reference SARS-CoV-2 Database

**Figure 1:**
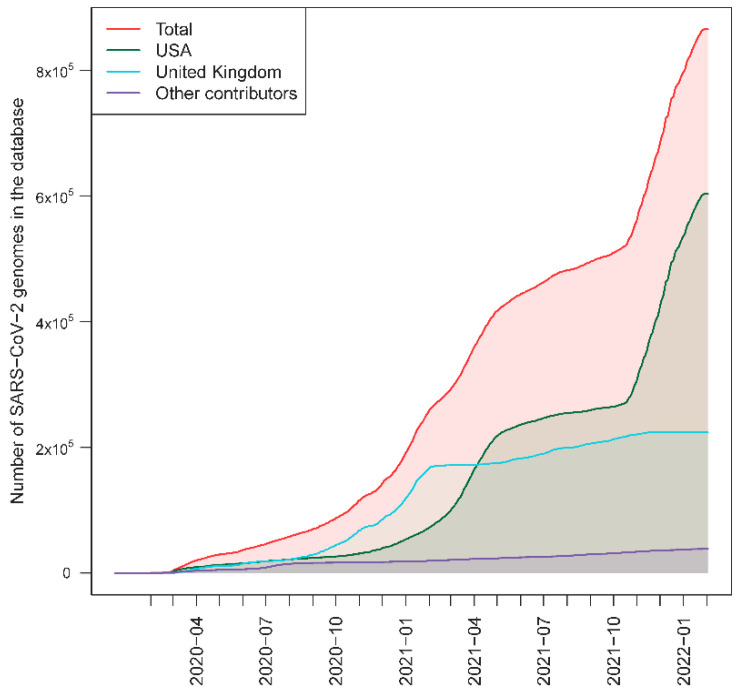
Accumulation of SARS-CoV-2 genomes in the GenBank database over time and the two main contributors, the UK and USA; the rest of the contributors have been grouped in a single category.

We used a database that contains >5.3M SARS-CoV-2 genomes. There are metadata associated to most of the genomes, including sampling dates and country. Figure 1 shows that there was a notable increased sequencing effort during 2021, with the arrival of the B.1.1.7 (alpha) SARS-CoV-2 variant to the pandemic scenario [21,22]. The sequencing effort is clearly biased towards a few countries that provide the main proportion of the genomes to the database: ~95.6% of the database has been contributed by USA (69.7%) and UK (25.9%) combined (Table S1).

### 2.2. Estimating the Timeframe of a Queried Genome

We first employed UShER [23] to allocate a SARS-CoV-2 genome to a huge prebuilt global phylogeny that contains 3,987,049 tip branches and 847,000 nodes. Using a standard computer (Intel i5—8th gen, 32Gb RAM, single thread, spinning disks), it took 12 s to load the reference tree, 12 s to allocate the QG of interest in the phylogeny (provided as an VCF file) and 4 s to write the tree output (29 s in total).

We firstly examined the efficiency of the simplest models to estimate *t*_E-QG_. The best approach to the *t*_E-QG_ comes from using matched RGs in the database (RG = QG; median value: 0 [1st−2nd quartiles: −8–5.5]) (Table 1). However, the reference database contains matched RGs for 74.4% of the QGs; for those QGs that do not have matched RGs in the database it is necessary to incorporate mutational derivative and ancestral RGs to the computation of *t*_E-QG_. A correction based on β = 7 days improved the estimates for models 1-SMD (median: −1 [–10–12]), 2-SMD (median: −4 [–16–11]), 3-SMD (median: −5 [−20–11]), 3-SMA (median: 10.5 [−7–21]), and 1/2-SB (median: −1 [−15–14]); while a no correction (β = 0) yields better estimates for the other simple models, namely, 1-SMA (median: −4 [−18–3]), 2-SMA (median: −6 [−22–1]), and 2/1-SB (median: −1 [−16–10]).

**Table 1:** Estimates for sampling times of a queried SARS-CoV-2 genome. We report the median of the individual values and the interquartile ranges of the medians. ‘Missing’ refers to the percentage of genomes that do not satisfy the necessary conditions for each model, e.g., not all the QG have matched RGs in the phylogeny. The outlier values represent the proportion of estimations with values above 3rd quartile +1.5 IQR (interquartile range), or below 1st quartile +1.5 IQR quartile +1.5 IQR. In bold are the best corrections within models. In brackets: number of different substitutions between the RG and the QG/Beta value coefficient. Abbreviations: NC = no correction; 1-SMD = RG is a 1-step mutational derivative from QG; 1-SMA = RG is a 1-step mutational ancestor from QG; 1/1-SB = RG and QG are in sister branches of 1-step mutational length each; 2-SMD = RG is a 2-step mutational derivative from QG; 2-SMA = RG is a 2-step mutational ancestor from QG; 3-SMD = RG is a 3-step mutational derivative from QG; 3-SMA = RG is a 3-step mutational ancestor from QG; 2/1-SB = RG and QG are in sister branches, the length of the RG branch is 2 substitutions and the length of QG is 1 substitution; 1/2-SB = RG and QG are in sister branches, the length of the RG branch is 1 substitution and the length of QG is 2 substitution; 1-M_SM = RG match or is a 1-step mutational ancestral or derivative from QG; 1/2-M_SM = RG match or is a 1 or 2-step mutational ancestral or derivative from QG; 1/2/3-M_SM = RG match or is a 1, 2 or 3-step mutational ancestral or derivative from QG. See also Figure 2.

Models *	Correction	Median	1st Quartile	2nd Quartile	Missing	Outliers
Simple models						
**RG = QG**	**NC**	**0**	**−8**	**5.5**	**74.4**	**10.5**
**1-SMD (1/1)**	NC	6	−3	19	79.7	5.8
	**b = 7**	**−1**	**−10**	**12**	**79.7**	**5.8**
	b = 16	−10	−19	3	79.7	5.8
**1-SMA (1/1)**	**NC**	**−4**	**−17.5**	**3**	**71.5**	**5.8**
	b = 7	−11	−24.5	−4	71.5	5.8
	b = 16	−20	−33.5	−13	71.5	5.8
**2-SMD (2/2)**	NC	10	−2	25	83.3	4.6
	**b = 7**	**−4**	**−16**	**11**	**83.3**	**4.6**
	b = 16	−22	−34	−7	83.3	4.6
**2-SMA (2/2)**	**NC**	**−6**	**−22**	**1**	**69.3**	**5.2**
	b = 7	8	−8	15	69.3	5.2
	b = 16	26	10	33	69.3	5.2
**1/1-SB (2/0)**	**NC**	**2**	**−10**	**14**	**75.5**	**5.1**
**3-SMD (3/3)**	NC	16	1	32	87.3	5.2
	**b = 7**	**−5**	**−20**	**11**	**87.3**	**5.2**
	b = 16	−32	−47	−16	87.3	5.2
**3-SMA (3/3)**	NC	−10.5	−28	0	73.0	4.5
	**b = 7**	**10.5**	**−7**	**21**	**73.0**	**4.5**
	b = 16	37.5	20	48	73.0	4.5
**2/1-SB (3/1)**	**NC**	**−1**	**−16**	**10**	**74.1**	**4.1**
	b = 7	6	−9	17	74.1	4.1
	b = 16	15	0	26	74.1	4.1
**1/2-SB (3/1)**	NC	6	−8	21	79.6	4.7
	**b = 7**	**−1**	**−15**	**14**	**79.6**	**4.7**
	b = 16	−10	−24	5	79.6	4.7
Mixed models						
**1-M_SM**	**NC**	**0**	**−8**	**5.5**	**74.4**	**10.5**
	b = 7	−0.5	−12.5	5.5	53.2	7.5
	b = 16	0	−11	7	53.2	7.4
**1/2-M_SM**	**NC**	**1**	**−9**	**9**	**53.2**	**7.2**
	b = 7	−1.5	−15	5	31.4	6
	b = 16	2	−9	13	31.4	4.6
**1/2/3-M_SM**	**NC**	**7**	**−5**	**24**	**31.4**	**2.4**
	b = 7	−3	−17	5	16.5	5.6
	b = 16	4	−9	15	16.5	4

Next, we evaluated mixed models; those that admit a mixed set of RGs (e.g., matched genomes, 1-step mutational derivatives, etc.). A very clear trend emerged in these models: considering correction *κ* = −β× #{number of substitutions relative to QG} with β value set as 0, 7, and 16, we observed that in all cases the value β = 0 yields the closest *t*_E-QG_ (Table 1). The model that considers matches or 1-step mutational ancestral or derivative from QG (1-M_SM) provides a median value of 0 (−8–5.5); the model that incorporates 2-step mutational ancestral or derivative RGs has comparable values (1/2-M_SM; median: −1 [−9–9]); this is also the case for the model that incorporates 3-steps mutational ancestral or derivative RGs (1/2/3-M_SM; median: -3 [−17–5]; Table 1 and Figure 3.

It is remarkable that the use of β = 16 days worsens the estimates in all the models (Table 1). Moreover, it is a general trend that *t*_E-QG_ values increase their variability (larger interquartile ranges) as more phylogenetically distant genomes are considered, even though the median value remains very close to the *t*_R-QG_; e.g., estimates of *t*_E-QG_ that incorporate 1-step mutational RGs are more precise and have less variability than those that incorporate 3-step mutational RGs; Figure 3 and Figure 4.

Finally, we also evaluated the performance of using the corrected *t*_R-RS_ values obtained from Chronumental (Table S2). Overall, Chronumental corrected times do not improve estimates of *t*_E-QG_ with respect to estimates obtained from non-corrected *t*_R-RG_ (Table 1; see also Supplementary Note S1).

### 2.3. Error Associated to the t_E-QG_ Estimates

The error associated to the estimates can be examined by comparing the *t*_E-RG_ to the *t*_R-RG_. Thus, Figure 5 shows the distribution of |*t*_E-RG_–*t*_R-RG_| values for the most common models in Table 1. For the simplest model that only considers RGs that match the QG (RG = QG), the Percentile 50 (P50) of the estimates has an average error or 6.5 days, the P75 = 15, and P90 = 28 (Figure 5). The error estimates are only slightly higher when using more complex models that consider RGs differing by 1, 2 or 3 mutations, e.g., P50 = 8 (1-M_SM), P50 = 9 (1/2-M_SM), and P50 = 10.5 (1/2/3-M_SM) (Figure 5).

It is also notable that the interquartile ranges (IQR) for the *t*_E-RG_ estimates are very low for the bulk of the cases (Figure S3). For the simple model RG = QG, the IQR percentiles are P50 = 5, P75 = 12.75, and P90 = 22; and these values are comparable when introducing more complex models; e.g., P50 = 7 (1-M_SM), P50 = 7 (1/2-M_SM), and P50 = 11 (1/2/3-M_SM); Figure S3.

### 2.4. The Effect of Database Geographic Coverage

Most of the SARS-CoV-2 genomes in the database were sampled in USA and UK (Figure 1). Therefore, it would be somehow expected that inferences of the *t*_E-QG_ would be more precise for QGs sampled in USA and UK than for QGs sampled elsewhere. Contrary to expectations, the values obtained for QGs of non-USA/UK samples are perfectly comparable to those in USA and UK regardless the model considered (Figure S1). For instance, the values for the 1M-SM model are median: −0.1 [−10.1–4.0] for USA, 0 [−5.1–6.9] for UK, and −0.1 [−5.2–5.1] for other countries.

## 3. Discussion

Recent publications analyzed post-mortem persistence of SARS-CoV-2 [24,25,26,27,28], but these studies did not focus on PMI. The potential forensic interest of using the methodology developed in the present study rests on its power to obtain estimates of PMI from one month before death backwards—an interval for which traditional techniques provide only very weak qualitative estimates. The VMCD method only needs to retrieve and sequence (using very well-established NGS techniques and protocols) the RNA of the targeted virus (the SARS-CoV-2 in our proof-of-concept simulations). Then, the sequence can be easily processed as indicated in the present study (Figure 6); then a narrow estimate of the viral infection moment can be obtained in seconds.

Previous studies have demonstrated that the RNA of a virus can be sequenced in very old specimens. White et al. [29] demonstrated that postmortem human brain tissue collected by tissue hospital banks over decades can contain high quality material for RNA analysis. RNA can also be retrieved from historical and ancient samples. For example, Smith et al. [30] retrieved archaeological RNA genome of Barkey Stripe Mosaic Virus (BSMV) isolated from a barley grain ~750 years of age. Guzmán-Solís et al. [31] analyzed ancient viral genomes in the context of the transatlantic slave trade. Recent studies have retrieved ancient RNA from samples dating back to >14,000 years [32]. These studies demonstrate the potential of modern lab techniques to retrieve RNA from samples that could be of interest in a legal medicine context [33].

The present study has used the SARS-CoV-2 model as a proof of concept. The SARS-CoV-2 genome database has been employed for convenience because of its large sample size and the availability of the metadata associated to the genomes. There are now millions of SARS-CoV-2 genomes available that have been generated during the COVID-19 pandemic (Figure 6), and the database is continuously growing. However, the VMCD methodology might be applied to any other pathogen, provided that there exists a large genome database for it. The method takes advantage of the emergence of large genome reference databases and new computational phylogenetic approaches that allow to rapidly build phylogenetic trees using millions of genome sequences of a pathogen of interest [23,34]. The COVID-19 pandemic has demonstrated that it is technically possible to generate enormous genome databases of microorganisms in short time periods: from 2020 to mid 2021, laboratories worldwide generated >5.3M entire genome sequences of circulating SARS-CoV-2 around the world (~6.2% of them in 2020 and ~69.5% in 2021; the remaining in 2022); most are freely available in public databases (GISAID and GenBank). Therefore, it is currently plausible to envision the emergence of new pathogen genome databases in a near future given the technological facilities available, the reduced costs of Next Generation Sequencing (NGS) techniques and, most importantly, the growing ambition of international health agencies to prevent and monitor emerging pandemic threats. According to WHO, antimicrobial resistance (AMR) is one of the top 10 global public health threats facing humankind and there are several viruses with a high potential to originate future pandemics. In this context, sequencing viral genomes is the method of choice to keep adequate traceability of circulating viruses and bacteria. Furthermore, it is important to note that there are in fact large genome databases for other pathogens (e.g., influenza, zika and enterovirus) for which there are already large phylogenetic developments (see, e.g., https://nextstrain.org, accessed on 17 May 2022). This global scenario offers an opportunity to investigate the potential applications of these pathogen databases in a legal medicine context that goes well beyond their obvious microbiological and epidemiological interest.

We have observed that the developed VMCD method works well with a variety of phylogenetic topologies; each single estimate is carried out on a phylogenetic tree that is unique because it depends on the amount and specific features of the RGs available for each QG. We have also seen empirically that β = 0 days per substitution difference between a RG and the QG works satisfactorily for many of the models tested, especially when considering ancestral scenarios. In turn, β = 7 is the best choice for the analysis of models with predominant derivative sequences. In any case, these correction works much better than β = 16, the value deduced from evolutionary mutation rates. The rationale behind this discrepancy could be related to the different evolutionary timescales: while the estimates carried out in the present study are obtained from short-term timescales (of a few days or weeks), substitution (evolutionary) rates are generally obtained with a long temporal perspective (of years), a time period that allows for a more efficient action of purifying natural selection. These discrepancies have already been reported in other organisms, see, e.g., mtDNA in humans [35].

The use of a VMCD method to indirectly estimate the PMI and other forensic applications has the following disadvantages:

*(i)*  The RNA of a microorganisms of interest might not be present, or be too degraded, in the corpse or the biological sample of forensic interest.
*(ii)*  A large reference genome database is always mandatory for the microorganism of interest.
*(iii)*  The method is probably sensitive to missing or incorrect data (in both the reference database and the interrogated genomes); however, the simulations carried out by Turaknhia et al. [23] in UShER suggested that time estimates could experience only minor deviations.

Among the main advantages of this method are:

*(i)*  It has potential for the estimation of time intervals running from about one month in the past to a (very) distant past (of years ago), which is the time range not covered by current methods (e.g., present-day methods for the estimation of the PMI only work for periods of just a few hours/days in the past).
*(ii)*  It is highly precise because even for several years old specimens it may allow to obtain short error estimates (measured as IQRs) of only a few days/weeks; moreover, the IQRs are approximately constant if the reference database keeps a considerable sample size over time (e.g., in the order of the one employed in the present study).
*(iii)*  It would work for symptomatic or asymptomatic clinical cases [36] and it does not depend on the severity and body tissue [37] analyzed because the only information that is needed for the analysis is the genome sequence of the microorganism, independently of the clinical manifestations of the infected individual.
*(iv)*  The procedure performs well in circumstances where the QGs do not have a well-represented local reference genome database. A possible explanation for this may be that the SARS-CoV-2 phylogenetic tree is so large that the deficient contribution of some countries to the database might be phylogenetically compensated by the large contribution of a few countries.
*(v)*  It is cost effective because it would only require sequencing the whole genome of the microorganism using well-established NGS techniques (currently available in, e.g., most hospital settings and research institutions).
*(vi)*  Theoretically, the SARS-CoV-2 VMCD method would work even if more than one strain is present because all the infected strains in an individual should have comparable ‘evolutionary times’ (moreover, note that usually it is the consensus genome sequence of the virus that is reported in an individual and in genome repositories).
*(vii)*  The ultrafast sample placement of QGs in a preexisting phylogeny provides an agile and computationally easy environment for real casework without specialized personnel.
*(viii)*  The method is not sensitive to environmental factors (beyond the effect these factors could have in degrading the genetic material and precluding its sequencing).
*(ix)*  More than one pathogen could be used to obtain independent estimates of infection times.

There are a few relevant limitations of the present study. First, we did not thoroughly investigate alternative procedures to infer *t*_E-QG_ from the closest phylogenetic reference genomes; however, the approach of using the median and interquartile ranges of the *t*_R-RG_ values to estimate the *t*_E-QG_ seems to perform satisfactorily (Table 1), and even better than the stochastic gradient descent method implemented in Chronumental. In any case, the present study could be extended in several aspects, including the use of other phylogenetic approaches, a focus on other pathogens, measuring the impact of sequencing errors and missing sequence data, etc. Second, the procedure tested in the present study did not consider that viral variants [20] might evolve at different rates [38]. Related to this, the rate of viral evolution in immunocompromised patients (at least in those with primary defects) has been shown to be faster than in the general population ([39,40]). A more sophisticated method to correct time estimates could consist of calibrating the molecular clock by identifying those genome sites that are under accelerated evolution. However, the results of the present study suggest that error rates are very low in the vast majority of the QGs tested, and also, that the best estimates are obtained when avoiding the use of molecular clock corrections. This suggests that possible deviations from a constant molecular clock are compensated by the power provided by the use of a number of phylogenetic RGs for time estimates, instead of relying on point estimates for the QGs (as done by, e.g., Chronumental).

Other questions remain to be further investigated. For instance, it would be relevant to know more about the postmortem stability of a virus in a corpse. Prescott et al. [41] indicated that viable Ebola virus could be isolated seven days post-euthanasia, and the viral RNA could be detectable for 10 weeks. In fact, it is possible to detect RNA from a virus in human remains dated to several hundred [42] or even thousands of years [43,44]. A recent case report by Bonelli et al. [26] showed the persistence of SARS-CoV-2 viral genome in nasopharyngeal swabs performed on a drowned man who was completely asymptomatic when he was alive, up to 41 days after death. These authors highlighted the importance of postmortem swabs in all autopsy cases, a suggestion that would make perfect sense in the context of the present study. In the same vein, Gabbrielli et al. [45] detected the SARS-CoV-2 genome in the corpse of an exhumed infected person one month after her death. It is also important to note that viruses usually replicate inside living cells; thus, a virus should (theoretically) slow down replication after death [28], and therefore it should not accumulate substantial mutational changes (at least to a level affecting the consensus genome sequence) after death of the host; this issue should, however, be investigated further in the same line as in Grassi et al. [28]. For the same reason, a postmortem SARS-CoV-2 infection should not have a relevant impact in a corpse from the point of view of viral load because the new virions should not be able to replicate to a detectable and significant amount. Another interesting question of forensic interest would be on the tissues that can be infected by a virus. In the case of the SARS-CoV-2, although the preferable site of infection is the respiratory track, the virus has been detected in kidney, liver, heart autopsy tissue samples [46] and many other tissues of the urinary tract (e.g., kidney), reproductive system (e.g., prostate, testicle), hematological tissues (e.g., blood, bone marrow), endocrine system (e.g., thyroid gland), etc. [47,48].

The results of the present study are clear in showing that dating viral genomes can be straightforward and highly precise if a proper database is available, and this method could assist in the resolution of cases of legal medicine interest, from criminalistics to casework related to samples stored in hospital settings. Traditional phylogenetic methods for dating have been conceived to date nodes of a tree (while the target of the present study is a specific QG), are computationally very demanding, and require high expertise in evolutionary theory and complex decision-making on demographic and other evolutionary parameters. It was not within the scope of the present study to evaluate the best possible phylogenetic and analytical method to obtain time estimates, but to provide solid evidence demonstrating that a simple and rapid phylogenetically inspired method could benefit routine forensic casework, complementing and improving present-day techniques.

## 4. Material and Methods

### 4.1. The SARS-CoV-2 Genome Database

A total of 20,000 sequences were randomly selected from GenBank to be used as queried genomes (QGs), simulating a real scenario of sampling a SARS-CoV-2 genome from a corpse or a biological source of interest. In these cases, we know beforehand their recorded sampling times (recorded sampling time of a QG: *t*_R-QG_). Details on the countries represented in the database and the random sampling are provided in Table S1. The impact of overrepresented countries in the database, namely, USA and UK (Table S2), has been analyzed in a separate section, by structuring the dataset in three groups, namely USA, UK and the category ‘others’ that includes samples from the remaining countries.

### 4.2. Phylogenetic Allocation of Genomes into the SARS-CoV-2 Tree

We employed the tool UShER [23] to allocate a QG to a global phylogenetic tree. For this purpose, we used the same SARS-CoV-2 genome database considered in UShER (downloaded on 13 February 2022). The usual SARS-CoV-2 reference sequence (GenBank code: MN908947.3) was used for alignment and annotation. This database contains a total of 5,322,161 genomes; all of them deposited in GenBank. The associated metadata for these genomes was downloaded (https://hgdownload.soe.ucsc.edu/goldenPath/wuhCor1/UShER_SARS-CoV-2/ accessed on 17 May 2022), and includes information on sampling date and location, GenBank accession number, sequence length, pangolin, Nextstrain and UShER clade.

### 4.3. Basic Time Estimates for Queried Genomes

Our aim is to estimate when sampled QGs were circulating in a given population (estimated circulating time for the QG of interest: *t*_E-QG_) by investigating the sampling dates of their closest phylogenetically related reference genomes in the tree (recorded time of a given RG: *t*_R-RG_); Figure 6. Ideally, *t*_E-QG_ should match *t*_R-QG_ as closely as possible to. The overall process is as follows: once a QG is allocated to the global phylogeny with UShER, the 40 RGs phylogenetically closest to the QG are retained. Only those that differ from the QG by less than four substitutions are kept. This threshold is established for practical purposes; but we empirically proved that the use of a larger number of RGs (which usually have more mutational differences from the QG) worsens the estimates (see results). The *t*_E-QG_ values are estimated as the mean (together with their corresponding standard deviations) or median (together with their corresponding interquartile ranges) of the *t*_R-RG_. Mean and median values are very similar in all the estimates; however, we chose the median because it is more robust than the mean to measure central tendency (being high inelastical, in contrast to the mean); and quartile ranges to measure statistically dispersion (more appropriate for data that is not normally distributed). Note that, unless otherwise specified, the median values referred to in the text (Table 1) are the median of the individual median values and the interquartile ranges of the medians. We also demonstrate that this estimates based on the median of the *t*_R-RG_ performs better than the more sophisticated and computationally demanding method implemented in Chronumental [34].

The efficiency of the VMCD method can be evaluated simply by examining how close *t*_E-QG_ are to *t*_R-QG_ values. To avoid over-fitting of the *t*_E-QG_ values, the 20,000 genomes from the USHeR subset used as QGs were excluded from the reference database (Figure 6).

### 4.4. Corrected Time Estimates for Queried Genomes Using the Molecular Clock

**Figure 2:**
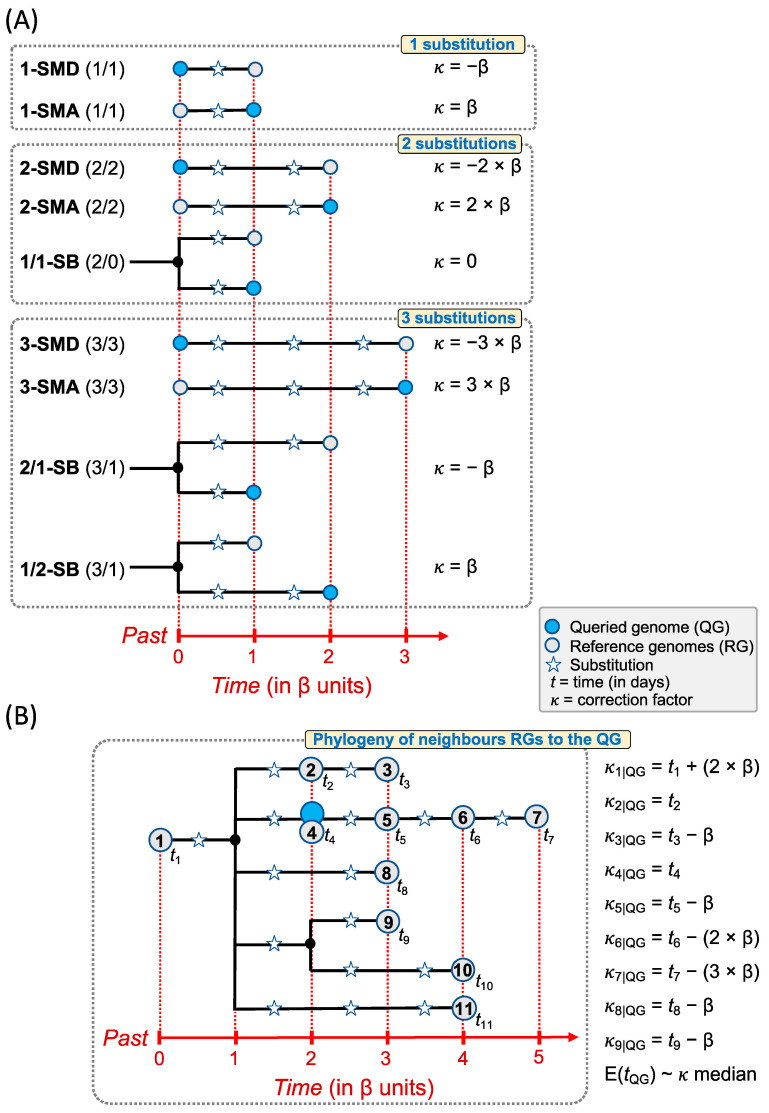
Diagram showing the procedure for sampling time corrections for the reference genomes (RG) of a given queried genome (QG). (**A**) The time of a QG (*t*_E-QG_) can be estimated using sampling dates of phylogenetically close RGs (t_R-RG_). The figure shows all the most parsimonious evolutionary possibilities connecting a QG to an RG when differing by 1, 2, or 3 substitutions. A way to estimate the age of the QG is to apply a correction factor 𝜅 to the neighbor RGs; this factor depends on the number of substitutions separating the QG and the RG, the shape of the phylogeny and the time needed to accumulate one substitution in circulating SARS-CoV-2 genomes (β). (**B**) Diagram showing the procedure to estimate the age of a queried genome (QG; *t*_QG_) using sampling dates of RGs from a hypothetical phylogeny of closely related genomes. Only RGs differing by 0, 1, 2, 3 substitutions with the QG are used for the inference; namely: RGs = {#1, #2, #3, #4, #5, #6, #7, #8, #9}. The correction factor 𝜅 is computed on all possible genome pairs RG|QG. We consider the median as the best estimate for the individual values.

**Figure 3:**
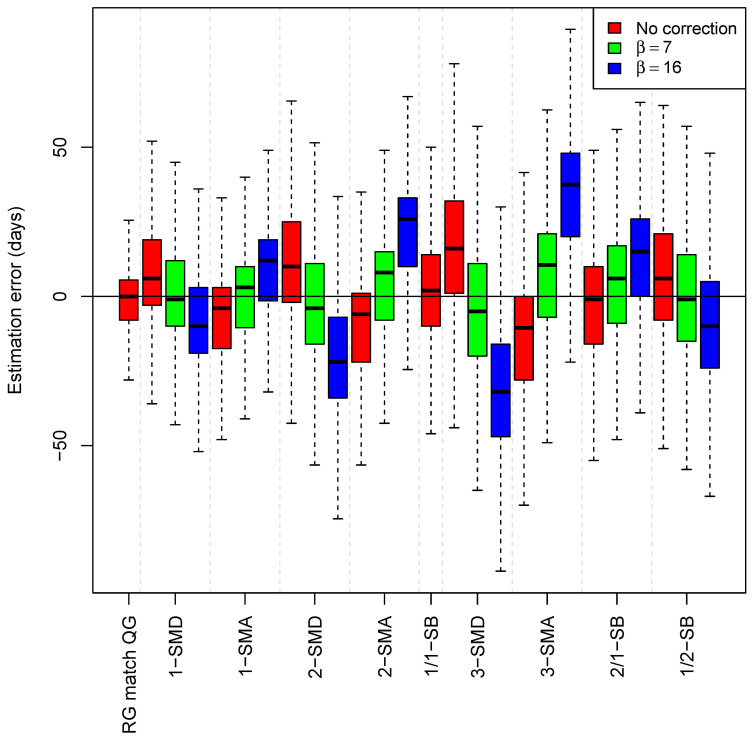
Timeframes (median values for the time of a QG (*t*_E-QG_) and interquartile ranges) for QGs in simple phylogenetic models. β = time correction by a single substitution; 1-SMD = RG is a 1-step mutational derivative from QG; 1-SMA = RG is a 1-step mutational ancestor from QG; 1/1-SB = RG and QG are in sister branches of 1-step mutational length each; 2-SMD = RG is a 2-step mutational derivative from QG; 2-SMA = RG is a 2-step mutational ancestor from QG; 3-SMD = RG is a 3-step mutational derivative from QG; 3-SMA = RG is a 3-step mutational ancestor from QG; 2/1-SB = RG and QG are in sister branches, the length of the RG branch is 2 substitutions and the length of QG is 1 substitution; 1/2-SB = RG and QG are in sister branches, the length of the RG branch is 1 substitution and the length of QG is 2 substitutions.

**Figure 4:**
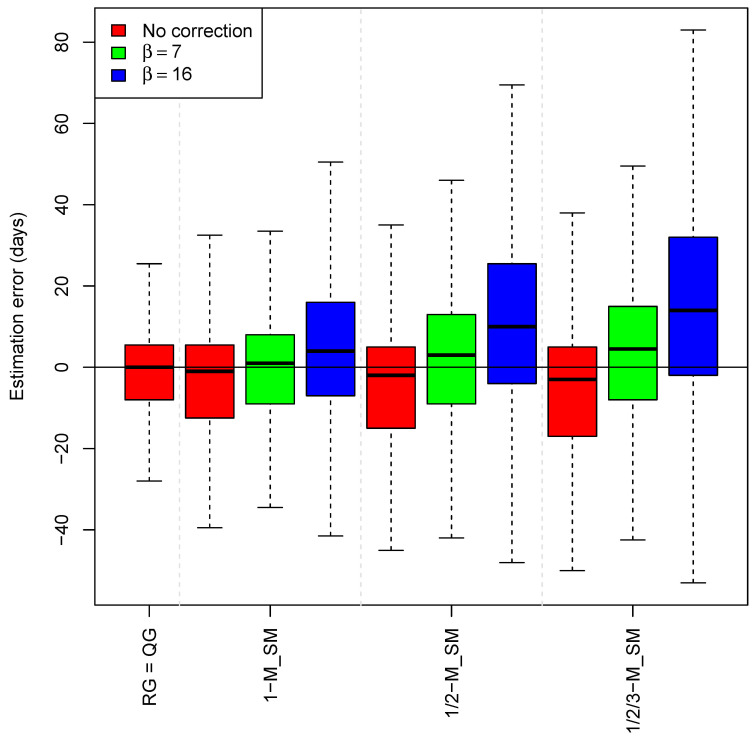
Timeframes (median values for the time of a QG (*t*_E-QG_) and interquartile ranges) for QGs in mixed phylogenetic models. β = time correction by a single substitution; 1-M_SM = RG match or is a 1-step mutational ancestral or derivative from QG, 1/2-M_SM = RG match or is a 1 or 2-step mutational ancestral or derivative from QG; 1/2/3-M_SM = RG match or is a 1, 2 or 3-step mutational ancestral or derivative from QG.

**Figure 5:**
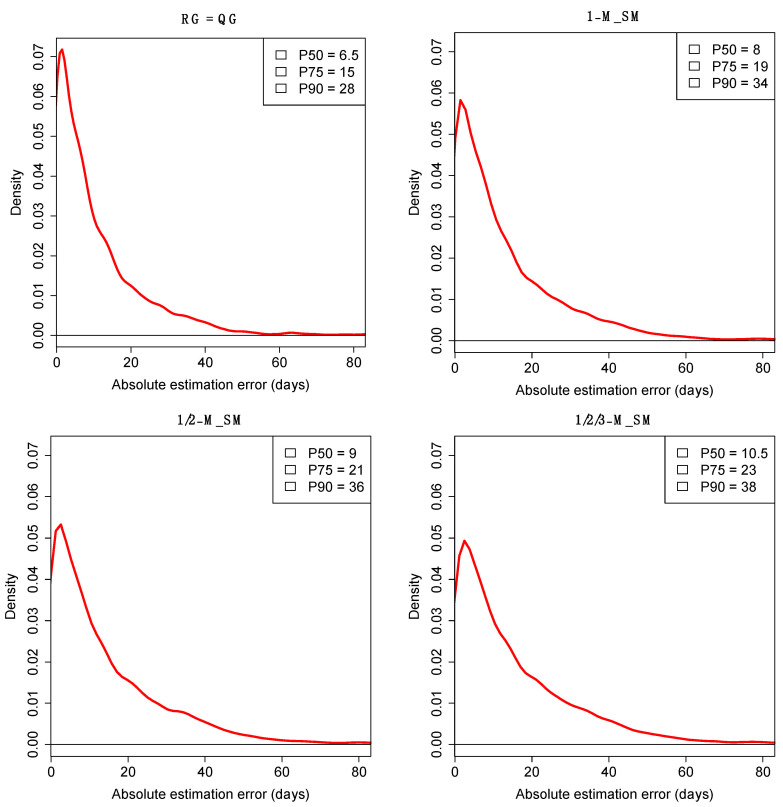
Errors (in absolute values) associated to the *t*_E-QG_ estimates under different simulation models. 1-M_SM = RG match or is a 1-step mutational ancestral or derivative from QG, 1/2-M_SM = RG match or is a 1 or 2-step mutational ancestral or derivative from QG; 1/2/3-M_SM = RG match or is a 1, 2 or 3-step mutational ancestral or derivative from QG; P50 = percentile 50; P75 = percentile 75, P90 = percentile 90.

**Figure 6:**
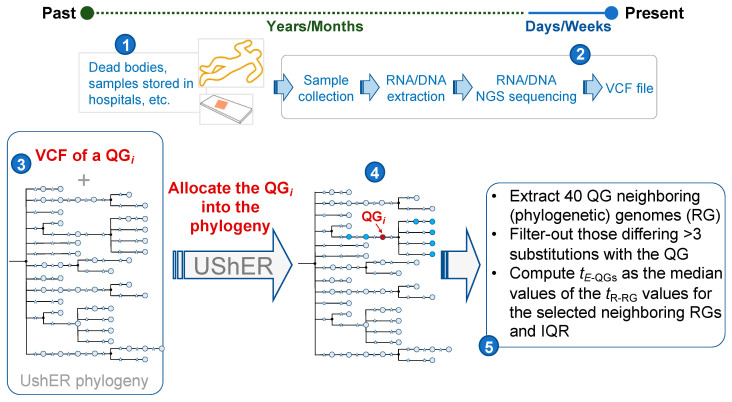
Diagram showing the VMCD procedure employed in the present project. The VMCD method allows to date queried viral genomes from about one month ago backwards (**1**). A biological sample is collected, and its RNA is extracted and sequenced (**2**); its sequence (QG) can be examined using available software (e.g., [19]), and, using an standard format (VCF) it is introduced in the pipeline (**3**) and allocated to its most likely node within the large SARS-CoV-2 phylogeny (**4**). Then, the neighboring RGs are retrieved, and their sampling times (t_R-RG_) processed, as indicated in the methodology section, to finally obtain the required t_E-QG_ value (**5**). The original database has 5,322,161 genomes that were used to build a huge UShER phylogenetic tree. A total of 20,000 genomes are extracted from the GenBank database and used as queried genomes (QG); the final reference database therefore contains 5,322,161 genomes. VCF: Variant Calling Format (typically used to process DNA/RNA sequences); *t*_E-QG_: estimated date of the sequence QG; *t*_R-RG_: estimated date of the sequence RG.

Aiming at narrowing the *t*_E-QG_, we also tested the performance of a correction factor (here denoted by *κ*) that accounts for the number of substitutions separating the QG from the RGs. The average time needed to accumulate a single substitution (here denoted by β) in circulating viral genomes can be calculated from the SARS-CoV-2 mutation rate (μ) and the length of the genome (L) as follows: β = 365/(μ × L). The rate of evolution of the SARS-CoV-2 has been estimated to be ~0.8 × 10^−3^–0.542 × 10^−3^ substitutions per site per year (s/s/y). Here, we used 0.8 × 10^−3^ s/s/y (Nextstrain; https://nextstrain.org/ncov/gisaid/global, accessed on 17 May 2022) and the average length of the SARS-CoV-2, which is 28.5Kb. Therefore, the mentioned values of μ and L make β ≈ 16 days [20]. However, we empirically observed that a β value of 0 or 7 days (depending on the simulated model) performs better than 16 days (see results below). When the QG matches a given RG (RG = QG), no correction factor is needed (*κ* = 0), so the best estimate for its *t*_E-QG_ would be the age of the identical (matched) RG or, if more than one, an average of their *t*_R-RG_. When two genomes differ in 1, 2, or 3 substitutions, *κ* can be easily calculated for the most common phylogenetic scenarios: briefly, these may consist of the RG and the QG differing by one, two, or three extra substitutions (models 1-SMD, 2-SMD, and 3-SMD, respectively) or differing by one, two, or three substitutions less (model 1-SMA, 2-SMA, and 3-SMA, respectively) (see possible phylogenetic trees in Figure 2; more details in Supplementary Note S1, Table S3).

These simple scenarios can be mixed in several ways but, for pragmatical reasons, it is not within the scope of the present study to analyze all possible combinations. We evaluated situations where the set of close phylogenetic neighbors of a QG contain simultaneously (*i*) RGs that match or are 1-step mutational ancestral or derivative from QG (model M_1-SM), (*ii*) RGs that match or are 1- or 2-step mutational ancestral or derivative from QG (model M_1-2SM), and (*iii*) RGs that match or are 1-, 2- or 3-step mutational ancestral or derivative from QG (model M_1-2-3-SM).

For the sake of comparing different approaches to estimate *t*_E-QG_ we additionally evaluated the phylogenetic tool Chronumental [34]; this software allows us to obtain ‘time trees’ from large phylogenies and to assign ‘corrected’ sampling times to the RGs of interest.

In all cases, extreme values of sampling times were deduced from boxplots following usual procedures: values that fall at least 1.5 interquartile ranges below the first quartile, or at least 1.5 interquartile ranges above the third quartile.

We used R software (version 4.0.3) to carry out all the statistical analysis and graphic representations [49].

## Data Availability

The authors confirm that all data used in this study was obtained from free public repositories.

## Supplementary Materials

The following supporting information can be downloaded at: https://www.mdpi.com/article/10.3390/ijms232112899/s1. Ref. [50] is cited in Supplementary Materials.

## References

[1] Sampaio-Silva F., Magalhaes T., Carvalho F., Dinis-Oliveira R.J., Silvestre R. (2013). Profiling of RNA degradation for estimation of post mortem [corrected] interval. PLoS ONE.

[2] Scrivano S., Sanavio M., Tozzo P., Caenazzo L. (2019). Analysis of RNA in the estimation of post-mortem interval: A review of current evidence. Int. J. Leg. Med..

[3] Dell’Aquila M., De Matteis A., Scatena A., Costantino A., Camporeale M.C., De Filippis A. (2021). Estimation of the time of death: Where we are now?. Clin. Ter..

[4] Hu L., Xing Y., Jiang P., Gan L., Zhao F., Peng W., Li W., Tong Y., Deng S. (2021). Predicting the postmortem interval using human intestinal microbiome data and random forest algorithm. Sci. Justice.

[5] Johnson H.R., Trinidad D.D., Guzman S., Khan Z., Parziale J.V., DeBruyn J.M., Lents N.H. (2016). A Machine Learning Approach for Using the Postmortem Skin Microbiome to Estimate the Postmortem Interval. PLoS ONE.

[6] Metcalf J.L. (2019). Estimating the postmortem interval using microbes: Knowledge gaps and a path to technology adoption. Forensic Sci. Int. Genet..

[7] Ciaffi R., Feola A., Perfetti E., Manciocchi S., Potenza S., Marella G.L. (2018). Overview on the estimation of post mortem interval in forensic anthropology: Review of the literature and practical experience. Rom. J. Leg. Med..

[8] Suchard M.A., Lemey P., Baele G., Ayres D.L., Drummond A.J., Rambaut A. (2018). Bayesian phylogenetic and phylodynamic data integration using BEAST 1.10. Virus Evol..

[9] Kumar S. (2005). Molecular clocks: Four decades of evolution. Nat. Rev. Genet..

[10] Capodiferro M.R., Aram B., Raveane A., Rambaldi Migliore N., Colombo G., Ongaro L., Rivera J., Mendizabal T., Hernandez-Mora I., Tribaldos M. (2021). Archaeogenomic distinctiveness of the Isthmo-Colombian area. Cell.

[11] Schroeder H., Sikora M., Gopalakrishnan S., Cassidy L.M., Maisano Delser P., Sandoval Velasco M., Schraiber J.G., Rasmussen S., Homburger J.R., Avila-Arcos M.C. (2018). Origins and genetic legacies of the Caribbean Taino. Proc. Natl. Acad. Sci. USA.

[12] Brandini S., Bergamaschi P., Cerna M.F., Gandini F., Bastaroli F., Bertolini E., Cereda C., Ferretti L., Gomez-Carballa A., Battaglia V. (2018). The Paleo-Indian Entry into South America According to Mitogenomes. Mol. Biol. Evol..

[13] Gómez-Carballa A., Pardo-Seco J., Brandini S., Achilli A., Perego U.A., Coble M.D., Diegoli T.M., Álvarez-Iglesias V., Martinón-Torres F., Olivieri A. (2018). The peopling of South America and the trans-Andean gene flow of the first settlers. Genome Res..

[14] Gómez-Carballa A., Bello X., Pardo-Seco J., Martinón-Torres F., Salas A. (2020). Mapping genome variation of SARS-CoV-2 worldwide highlights the impact of COVID-19 super-spreaders. Genome Res..

[15] Gómez-Carballa A., Bello X., Pardo-Seco J., Pérez Del Molino M.L., Martinón-Torres F., Salas A. (2020). Phylogeography of SARS-CoV-2 pandemic in Spain: A story of multiple introductions, micro-geographic stratification, founder effects, and super-spreaders. Zool Res..

[16] Pardo-Seco J., Gomez-Carballa A., Bello X., Martinon-Torres F., Salas A. (2021). Pitfalls of barcodes in the study of worldwide SARS-CoV-2 variation and phylodynamics. Zool Res..

[17] Wu F., Zhao S., Yu B., Chen Y.M., Wang W., Song Z.G., Hu Y., Tao Z.W., Tian J.H., Pei Y.Y. (2020). A new coronavirus associated with human respiratory disease in China. Nature.

[18] Bar-On Y.M., Flamholz A., Phillips R., Milo R. (2020). SARS-CoV-2 (COVID-19) by the numbers. Elife.

[19] Bello X., Pardo-Seco J., Gomez-Carballa A., Weissensteiner H., Martinon-Torres F., Salas A. (2022). CovidPhy: A tool for phylogeographic analysis of SARS-CoV-2 variation. Environ. Res..

[20] Gomez-Carballa A., Pardo-Seco J., Bello X., Martinon-Torres F., Salas A. (2021). Superspreading in the emergence of COVID-19 variants. Trends Genet..

[21] Davies N.G., Abbott S., Barnard R.C., Jarvis C.I., Kucharski A.J., Munday J.D., Pearson C.A.B., Russell T.W., Tully D.C., Washburne A.D. (2021). Estimated transmissibility and impact of SARS-CoV-2 lineage B.1.1.7 in England. Science.

[22] Davies N.G., Jarvis C.I., Group C.C.-W., Edmunds W.J., Jewell N.P., Diaz-Ordaz K., Keogh R.H. (2021). Increased mortality in community-tested cases of SARS-CoV-2 lineage B.1.1.7. Nature.

[23] Turakhia Y., Thornlow B., Hinrichs A.S., De Maio N., Gozashti L., Lanfear R., Haussler D., Corbett-Detig R. (2021). Ultrafast Sample placement on Existing tRees (UShER) enables real-time phylogenetics for the SARS-CoV-2 pandemic. Nat. Genet..

[24] Beltempo P., Curti S.M., Maserati R., Gherardi M., Castelli M. (2021). Persistence of SARS-CoV-2 RNA in post-mortem swab 35 days after death: A case report. Forensic Sci. Int..

[25] Sablone S., Solarino B., Ferorelli D., Benevento M., Chironna M., Loconsole D., Sallustio A., Dell’Erba A., Introna F. (2021). Post-mortem persistence of SARS-CoV-2: A preliminary study. Forensic Sci. Med. Pathol..

[26] Bonelli M., Rosato E., Locatelli M., Tartaglia A., Falco P., Petrarca C., Potenza F., Damiani V., Mandatori D., De Laurenzi V. (2022). Long persistence of severe acute respiratory syndrome coronavirus 2 swab positivity in a drowned corpse: A case report. J. Med. Case Rep..

[27] Heinrich F., Meissner K., Langenwalder F., Puschel K., Norz D., Hoffmann A., Lutgehetmann M., Aepfelbacher M., Bibiza-Freiwald E., Pfefferle S. (2021). Postmortem Stability of SARS-CoV-2 in Nasopharyngeal Mucosa. Emerg. Infect. Dis..

[28] Grassi S., Arena V., Cattani P., Dell’Aquila M., Liotti F.M., Sanguinetti M., Oliva A., Gemelli against COVID-19 Group (2022). SARS-CoV-2 viral load and replication in postmortem examinations. Int. J. Leg. Med..

[29] White K., Yang P., Li L., Farshori A., Medina A.E., Zielke H.R. (2018). Effect of Postmortem Interval and Years in Storage on RNA Quality of Tissue at a Repository of the NIH NeuroBioBank. Biopreserv. Biobank.

[30] Smith O., Clapham A., Rose P., Liu Y., Wang J., Allaby R.G. (2014). A complete ancient RNA genome: Identification, reconstruction and evolutionary history of archaeological Barley Stripe Mosaic Virus. Sci. Rep..

[31] Guzman-Solis A.A., Villa-Islas V., Bravo-Lopez M.J., Sandoval-Velasco M., Wesp J.K., Gomez-Valdes J.A., Moreno-Cabrera M.L., Meraz A., Solis-Pichardo G., Schaaf P. (2021). Ancient viral genomes reveal introduction of human pathogenic viruses into Mexico during the transatlantic slave trade. Elife.

[32] Smith O., Dunshea G., Sinding M.S., Fedorov S., Germonpre M., Bocherens H., Gilbert M.T.P. (2019). Ancient RNA from Late Pleistocene permafrost and historical canids shows tissue-specific transcriptome survival. PLoS Biol..

[33] Calvignac-Spencer S., Dux A., Gogarten J.F., Patrono L.V. (2021). Molecular archeology of human viruses. Adv. Virus Res..

[34] Sanderson T. (2022). Chronumental: Time tree estimation from very large phylogenies. bioRxiv.

[35] Howell N., Smejkal C.B., Mackey D.A., Chinnery P.F., Turnbull D.M., Herrnstadt C. (2003). The pedigree rate of sequence divergence in the human mitochondrial genome: There is a difference between phylogenetic and pedigree rates. Am. J. Hum. Genet..

[36] Nioi M., Napoli P.E., Fossarello M., d’Aloja E. (2020). Autopsies and Asymptomatic Patients During the COVID-19 Pandemic: Balancing Risk and Reward. Front. Public Health.

[37] Gómez-Carballa A., Rivero-Calle I., Pardo-Seco J., Gómez-Rial J., Rivero-Velasco C., Rodriguez-Nunez N., Barbeito-Castineiras G., Pérez-Freixo H., Cebey-López M., Barral-Arca R. (2022). A multi-tissue study of immune gene expression profiling highlights the key role of the nasal epithelium in COVID-19 severity. Environ. Res..

[38] Tay J.H., Porter A.F., Wirth W., Duchene S. (2022). The Emergence of SARS-CoV-2 Variants of Concern Is Driven by Acceleration of the Substitution Rate. Mol. Biol Evol..

[39] Corey L., Beyrer C., Cohen M.S., Michael N.L., Bedford T., Rolland M. (2021). SARS-CoV-2 Variants in Patients with Immunosuppression. N. Engl. J. Med..

[40] Weigang S., Fuchs J., Zimmer G., Schnepf D., Kern L., Beer J., Luxenburger H., Ankerhold J., Falcone V., Kemming J. (2021). Within-host evolution of SARS-CoV-2 in an immunosuppressed COVID-19 patient as a source of immune escape variants. Nat. Commun..

[41] Prescott J., Bushmaker T., Fischer R., Miazgowicz K., Judson S., Munster V.J. (2015). Postmortem stability of Ebola virus. Emerg. Infect. Dis..

[42] Schuenemann V.J., Avanzi C., Krause-Kyora B., Seitz A., Herbig A., Inskip S., Bonazzi M., Reiter E., Urban C., Dangvard Pedersen D. (2018). Ancient genomes reveal a high diversity of *Mycobacterium leprae* in medieval Europe. PLoS Pathog..

[43] Maixner F., Krause-Kyora B., Turaev D., Herbig A., Hoopmann M.R., Hallows J.L., Kusebauch U., Vigl E.E., Malfertheiner P., Megraud F. (2016). The 5300-year-old *Helicobacter pylori* genome of the Iceman. Science.

[44] Furtwangler A., Neukamm J., Bohme L., Reiter E., Vollstedt M., Arora N., Singh P., Cole S.T., Knauf S., Calvignac-Spencer S. (2020). Comparison of target enrichment strategies for ancient pathogen DNA. Biotechniques.

[45] Gabbrielli M., Gandolfo C., Anichini G., Candelori T., Benvenuti M., Savellini G.G., Cusi M.G. (2021). How long can SARS-CoV-2 persist in human corpses?. Int. J. Infect. Dis..

[46] Delorey T.M., Ziegler C.G.K., Heimberg G., Normand R., Yang Y., Segerstolpe A., Abbondanza D., Fleming S.J., Subramanian A., Montoro D.T. (2021). COVID-19 tissue atlases reveal SARS-CoV-2 pathology and cellular targets. Nature.

[47] Sheehan S.A., Hamilton K.L., Retzbach E.P., Balachandran P., Krishnan H., Leone P., Goldberg G.S. (2021). Evidence that *Maackia amurensis* seed lectin (MASL) exerts pleiotropic actions on oral squamous cells to inhibit SARS-CoV-2 infection and COVID-19 disease progression. Cell Res..

[48] Deinhardt-Emmer S., Wittschieber D., Sanft J., Kleemann S., Elschner S., Haupt K.F., Vau V., Haring C., Rodel J., Henke A. (2021). Early postmortem mapping of SARS-CoV-2 RNA in patients with COVID-19 and the correlation with tissue damage. Elife.

[49] R core Team (2019). R: A Language and Enviroment for Statistical Computing.

[50] Sagulenko P., Puller V., Neher R.A. (2018). TreeTime: Maximum-likelihood phylodynamic analysis. Virus Evol..

