# Supplementary material for: A Timeframe for SARS-CoV-2 Genomes: A Proof of Concept for Postmortem Interval Estimations
Source: Int J Mol Sci. 2022 Oct 25;23(21):12899. doi: 10.3390/ijms232112899 (PMC9656715; doi:10.3390/ijms232112899)
Supplement: Supplementary file 1 [file ijms-23-12899-s001.zip › Supplementary Figures.pdf]

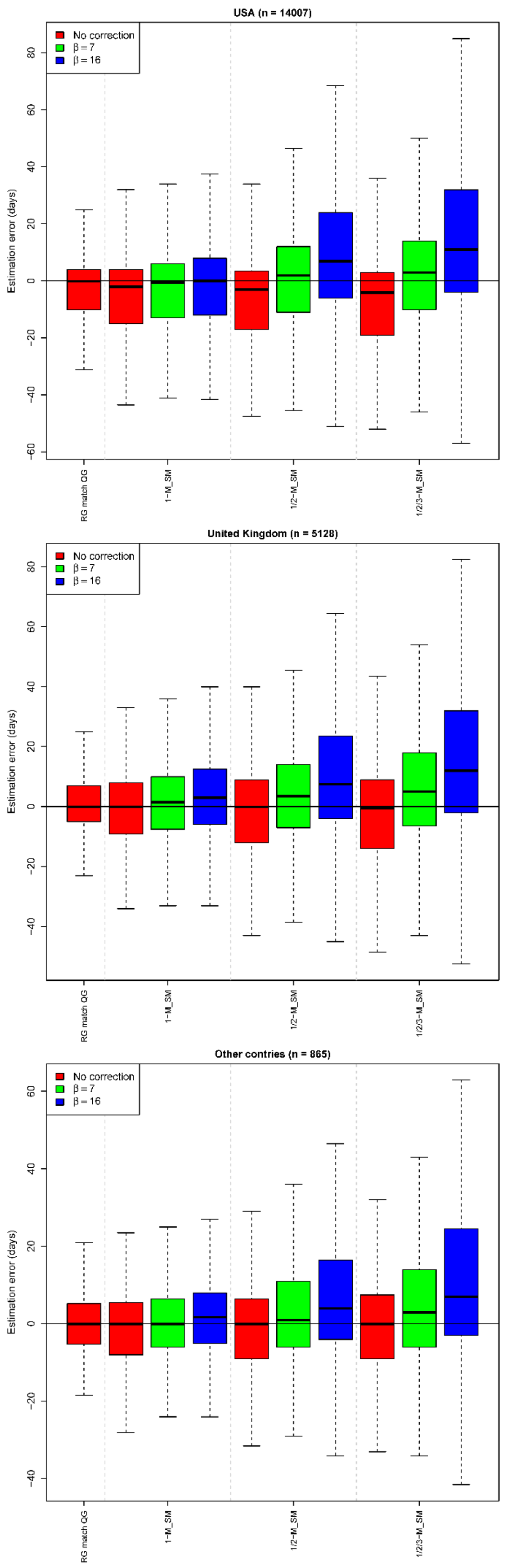

Figure S1. Timeframes (median values for the time of a QG ($t_{E\text{-}QG}$) and interquartile ranges) for QGs sampled in different countries. β = time correction by a single substitution; 1-M_SM = RG match or is a 1-step mutational ancestral

or derivative from QG, 1/2-M_SM = RG match or is a 1 or 2-step mutational ancestral or derivative from QG; 1/2/3-M_SM = RG match or is a 1, 2 or 3-step mutational ancestral or derivative from QG.

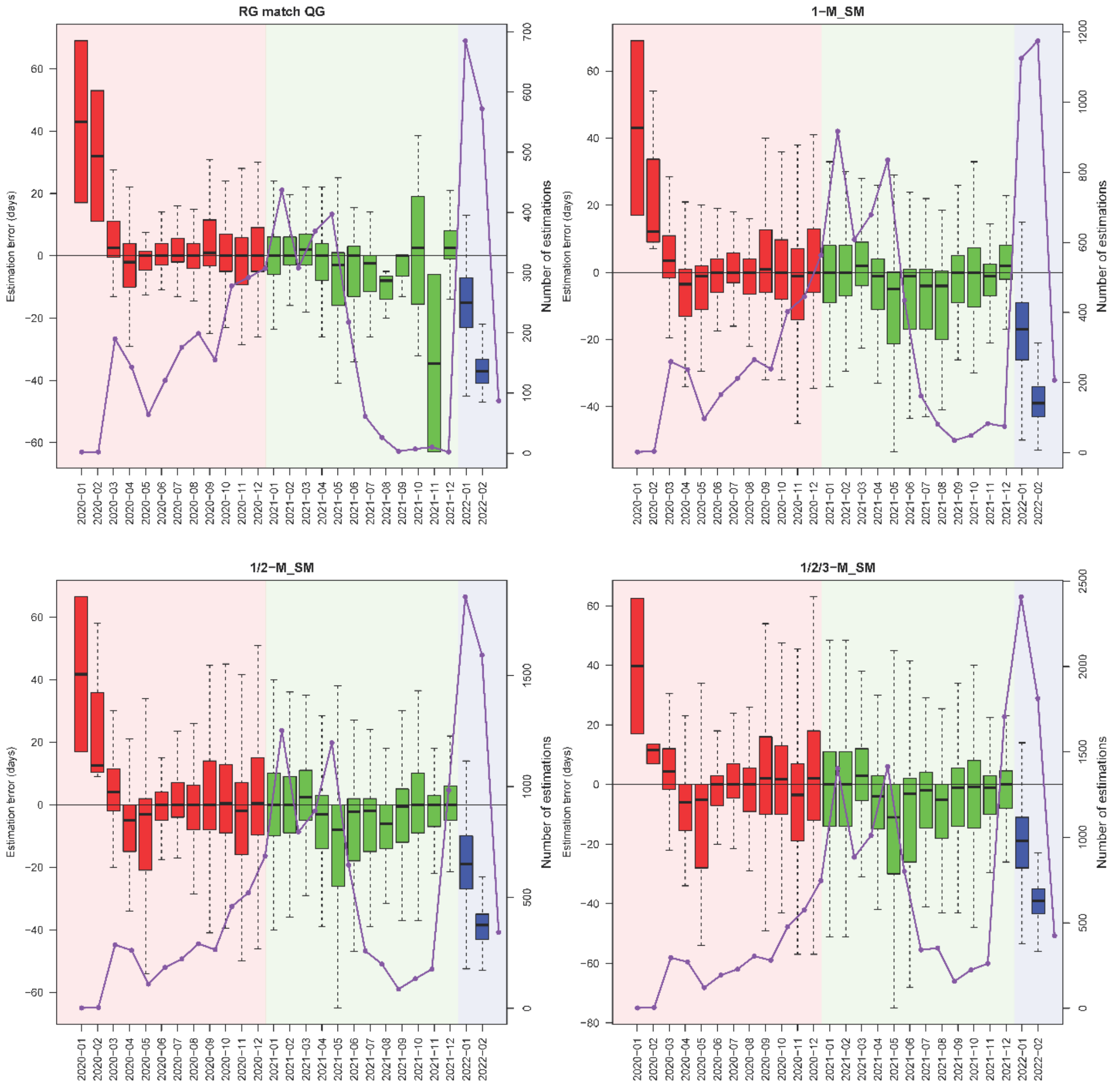

Figure S2. Interquartile ranges for the $t_{E\text{-}QG}$ and various simulation models. 1-M_SM = RG match or is a 1-step mutational ancestral or derivative from QG, 1/2-M_SM = RG match or is a 1 or 2-step mutational ancestral or derivative from QG; 1/2/3-M_SM = RG match or is a 1, 2 or 3-step mutational ancestral or derivative from QG; P50 = percentile 50; P75 = percentile 75, P90 = percentile 90.

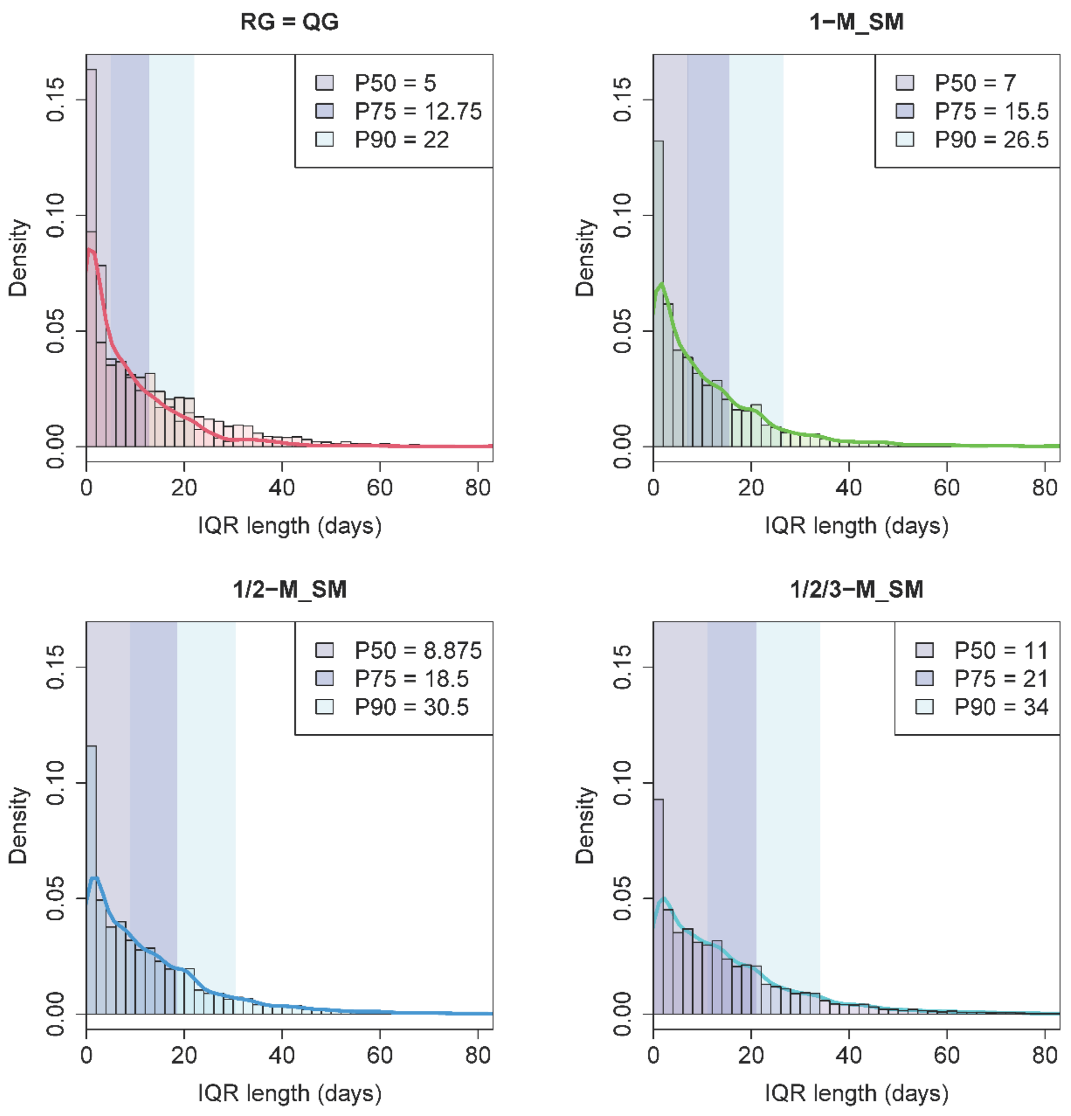

Figure S3. Distribution of IQR length (days) for each estimation and simulation model. 1-M_SM = RG match or is a 1-step mutational ancestral or derivative from QG, 1/2-M_SM = RG match or is a 1 or 2-step mutational ancestral or derivative from QG; 1/2/3-M_SM = RG match or is a 1, 2 or 3-step mutational ancestral or derivative from QG; P50 = percentile 50; P75 = percentile 75, P90 = percentile 90.
