# Supplementary material for: A Timeframe for SARS-CoV-2 Genomes: A Proof of Concept for Postmortem Interval Estimations
Source: Int J Mol Sci. 2022 Oct 25;23(21):12899. doi: 10.3390/ijms232112899 (PMC9656715; doi:10.3390/ijms232112899)
Supplement: Supplementary file 1 [file ijms-23-12899-s001.zip › Supplementary Note S1.pdf]

Supplementary Note S1

A timeframe for SARS-CoV-2 genomes: a proof of concept for postmortem interval estimations

Jacobo Pardo-Seco, Xabier Bello, Alberto Gómez-Carballa, Federico Martinón-Torres, Ignacio Muñoz-Barús, Antonio Salas

Supplementary Methodology

Correction factor *k* for the estimation of the $t_{E\text{-QG}}$

When two genomes (the RG and the QG) differ in 1, 2, or 3 substitutions, *k* can be calculated as follows, according to different simple phylogenetic scenarios (see also **Figure 2** in the main text and **Table S3** below):

(i) the RG and the QG differ by one substitution: if the RG contains one more substitution than the QG ($n_{RG} > n_{QG}$) this suggests a phylogenetic derived status of the RG (herein referred as model 1-SMD that states for: RG is a 1-step mutational derivative of QG), therefore $\kappa = -\beta$ days; on the contrary, if $n_{RG} < n_{QG}$ (model 1-SMA: RG is a 1-step mutational ancestor of QG), $\kappa = +\beta$ days;

(ii) the RG and the QG differ by two substitutions: $\kappa = -2 \times \beta$ days if $n_{RG} > n_{QG}$ and the total set of substitutions in the QG is included in the RG; that is QG $\subseteq$ RG (model 2-SMD: RG is a 2-step mutational derivative of QG); $\kappa = +2 \times \beta$ days if $n_{RG} < n_{QG}$ and the substitutions of the RG $\subseteq$ QG (model 2-SMA: RG is a 2-step mutational ancestor of QG); and $\kappa = 0$ days if $n_{RG} = n_{QG}$ and the substitutions of the RG $\not\subset$ QG and QG $\not\subset$ RG (both are sister branches in the tree);

(iii) the RG and the QG differ by three positions, $\kappa = -3 \times \beta$ days if $n_{RG} > n_{QG}$ and the full substitutions in the QG $\subseteq$ RG (model 3-SMD: RG is a 3-step mutational derivative from QG); $\kappa = +3 \times \beta$ days if $n_{QG} > n_{RG}$ and the full substitutions in the RG $\subseteq$ QG (model 3-SMA: RG is a 3-step mutational ancestor from QG); $\kappa = -\beta$ days if $n_{RG} > n_{QG}$ and not all the substitutions in the QG $\not\subset$ RG and RG $\not\subset$ QG (model 2/1-SB: RG and QG are in sister

branches, the length of the RG branch is 2 substitutions and the length of QG is 1 substitution); and $\kappa = +\beta$ days if $n_{QG} > n_{RG}$ and the substitutions in the QG $\not\subset$ RG and RG $\not\subset$ QG (model 1/2-SB: RG and QG are in sister branches, the length of the RG branch is 1 substitution and the length of QG is 2 substitutions).

Supplementary Results

Time estimates based on Chronumental

Overall, Chronumental corrected times (**Table S2**) do not improve estimates of $t_{E\text{-}QG}$ with respect to estimates obtained from non-corrected $t_{R\text{-}RG}$ (**Table 1** from main text), with only a few exceptions. Preliminary results using TreeTime [1] did not improve our results (e.g. larger IQR; data not shown), the method has the added disadvantage of requiring a more sophisticated pipeline. Other alternatives were not explored further for two main reasons: *i*) the phylogenetic software used in our pipeline was not the main focus of the present study (which is not on phylogenetic reconstructions and evolutionary issues), and *ii*) UShER provides such optimal results that it already satisfies the main aim of the project, namely, to provide a proof of concept on a new way to estimate narrow sampling times of a queried viral genome using a friendly and efficient pipeline.

Table S3. Values related to the models and example in **Figure 2**. S: substitutions

RG ID	Time	nº of *S*	nº extra *S*	nº missing *S*	Correction factor	Contributing time
RG = QG	t_1	0	0	0	0	$\kappa = t_1$
1-SMD	t_2	1	1	0	$-\beta$	$\kappa = t_2-\beta$
1-SMA	t_3	1	0	1	$+\beta$	$\kappa = t_3+\beta$
2-SMD	t_4	2	2	0	$-2\times\beta$	$\kappa = t_4-2\times\beta$
2-SMA	t_5	2	0	2	$t_5+2\times\beta$	$\kappa = t_5+2\times\beta$
1/1-SB	t_6	2	1	1	0	$\kappa = t_6$
3-SMD	t_7	3	3	0	$+3\times\beta$	$\kappa = t_7-3\times\beta$
3-SMA	t_8	3	0	3	$-\beta$	$\kappa = t_8+3\times\beta$
2/1-SB	t_9	3	2	1	$-\beta$	$\kappa = t_9-\beta$
1/2-SB	t_{10}	3	1	2	$+\beta$	$\kappa = t_{10}+\beta$
Example Figure 2B						
1	t_1	2	0	2	$+2\times\beta$	$\kappa_{1\|QG} = t_1+2\times\beta$
2	t_2	2	1	1	0	$\kappa_{2\|QG} = t_2$
3	t_3	3	2	1	$-\beta$	$\kappa_{3\|QG} = t_3-\beta$
4	t_4	0	0	0	0	$\kappa_{4\|QG} = t_4$
5	t_5	1	1	0	$-\beta$	$\kappa_{5\|QG} = t_5$
6	t_6	2	2	0	$-2\times\beta$	$\kappa_{6\|QG} = t_6-\beta$
7	t_7	3	3	0	$+3\times\beta$	$\kappa_{7\|QG} = t_7+3\times\beta$
8	t_8	3	0	3	$-\beta$	$\kappa_{8\|QG} = t_8-\beta$
9	t_9	3	2	1	$-\beta$	$\kappa_{9\|QG} = t_9-\beta$
10	t_{10}	4	3	1	-	û
11	t_{11}	4	3	1	-	û

References

1. Sagulenko, P.; Puller, V.; Neher, R.A. TreeTime: Maximum-likelihood phylodynamic analysis. *Virus Evol* **2018**, *4*, vex042, doi:10.1093/ve/vex042.
